# Comorbidities associated with HPV infection among people living with HIV-1 in the southeastern US: a retrospective clinical cohort study

**DOI:** 10.1186/s12879-020-4822-5

**Published:** 2020-02-14

**Authors:** Yuanfan Ye, Greer A. Burkholder, Howard W. Wiener, Russell Griffin, Stella Aslibekyan, Karen Fry, Ashraf Khan, Sadeep Shrestha

**Affiliations:** 1 Department of Epidemiology, School of Public Health, University of Alabama at Birmingham, Birmingham, AL 35242 USA; 2 Division of Infectious Diseases, School of Medicine, University of Alabama at Birmingham, Birmingham, AL 35242 USA; 3 Disease Control, Jefferson County Department of Health, Birmingham, AL 35233 USA

**Keywords:** HPV, HIV, LSIL, HSIL, Cancer

## Abstract

**Background:**

The southeastern US is an epicenter for incident HIV in the US with high prevalence of human papillomavirus (HPV) co-infections. However, epidemiologies of HPV-associated clinical conditions (CC) among people living with HIV-1 infection (PLWH) are not fully known.

**Methods:**

Electronic medical records (EMR) of PLWH attending one of the leading HIV clinics in the southeastern US between 2006 and 2018 were reviewed and analyzed. The retrospective study was nested within the University of Alabama at Birmingham HIV clinical cohort, which has electronically collected over 7000 PLWH’s clinical and sociobehavioral data since 1999. Incidence rates of HPV-related CC including anogenital warts, penile, anal, cervical, and vaginal/vulvar low- and high-grade squamous intraepithelial lesions (LSIL and HSIL) were estimated per 10,000 person years. Joinpoint regressions were performed to examine temporal changes in the trends of incident CC. All rates and trends were stratified by gender and race.

**Results:**

Of the 4484 PLWH included in the study (3429 men, 1031 women, and 24 transgender), we observed 1038 patients with HPV-related CC. The median nadir CD4 count (cells/uL) was higher in the HPV-condition free group than the case groups (*P* < 0.0001). Anogenital warts, anal LSIL, HSIL, and cancer were more likely to be diagnosed among HIV-infected men than women. White men presented more frequently with anal LSIL and anal and penile cancers than black men (*P* < 0.03). White women were also more likely to be diagnosed with cervical HSIL (*P* = 0.023) and cancer (*P* = 0.037) than black women.

**Conclusions:**

There were significant differences between gender and race with incidence of HPV-related CC among HIV patients. EMR-based studies provide insights on understudied HPV-related anogenital conditions in PLWH; however, large-scale studies in other regions are needed to generalize current findings and draw public health attention to co-infection induced non-AIDS defining comorbidities among PLWH.

## Background

Human papillomavirus (HPV) is a common sexually transmitted infection (STIs) in the general population and specifically among people living with HIV infection (PLWH) [1, 2]. While the infection is treatable, it has chronic sequelae including development of cancer [3]. In 2013–2014, the prevalence of anogenital HPV infection was estimated at 42.5% among US adults aged 18–59 years [4]. There were more than 14 million new diagnoses during this period [4]. Genital HPV infection is 1.5–2.5 times higher in HIV-positive (HIV+) women than HIV-negative (HIV-) women. HIV+ women have higher HPV acquisition, lower HPV clearance, higher incidence of low- (LSIL) and high- grade squamous intraepithelial lesions (HSIL) [5]. HPV prevalence is higher in HIV+ women than in HIV- for all cervical cytology group [6, 7]. Anal HPV infections among HIV+ women and MSM are 3 times higher than their HIV- counterparts [8–10].

HPV infection is common in the US, with over 80% of sexually active individuals being infected at least once during their lifetime [2]; however, most resolve on their own within 2 years. There are 12 types of low-risk HPV (LR-HPV) and at least 13 types of high-risk HPV (HR-HPV) [11]. LR-HPV cause warts and very mild cell changes in infected tracts, whereas persistent infection with HR-HPV cause LSIL and HSIL that can progress to cancer [12]. Over 99.7% of cervical is linked to HR-HPV infection [2]. Additionally, other anogenital cancers, including 95% of anal, 65% of vaginal, 50% of vulvar, and 35% of penile cancers are linked to HR-HPV infections [13]. HPV-associated cancers are diagnosed in 17,600 women and 9300 men every year in the US [14].

In the state of Alabama, racial disparities in new HIV infections and STDs have been documented; black women and men are highly susceptible to incident HIV [15]. While the epidemiology of HIV infection in Alabama is well-studied and reported, HPV-related clinical conditions (CC) among PLWH have not been comprehensively characterized. Neither the country nor the state of Alabama implements mandatory screening programs for anogenital HPV-related (excluding cervical cancer) conditions. Therefore, there is a substantial lack of knowledge of the comorbidities among PLWH. In this study, we retrospectively studied PLWH at risk of HPV- CC for over 12 years from the patients in the University of Alabama at Birmingham (UAB) 1917 Clinic, an academic institute with the largest HIV patient catchment in Alabama, and estimated HPV-CC incidence rates and comorbidity trends.

## Methods

### Study design and population

Medical charts of all patients attending the University of Alabama at Birmingham HIV clinic have been reviewed and electronic database was established (http://www.uab.edu/medicine/1917cliniccohort/). All relevant demographic, clinical and behavioral variables of patients between January 1st, 2006 and March 30th, 2018 were abstracted for this study. It is the largest HIV clinic in the state of Alabama with extensive referral network. The prospective clinic cohort has collected more than 7000 patients’ sociodemographic, psychosocial, and clinical information since its establishment in 1992 [16]. More than 3500 patients currently receive their routine HIV care from the clinic, representing 30% of all PLWH in the state [16]. This retrospective study was nested within the UAB 1917 Clinic Cohort and approved by the UAB Institutional Review Board.

In this study, eligible patients were patients who: 1) attended the clinic at least twice for receiving primary HIV care during the 12-year study period; and 2) were at least 18 years old at HIV diagnosis.

### Study variables

HPV-related CC were categorized into: anogenital warts and Bowen’s disease; anal LSIL and HSIL; cervical LSIL and HSIL; anal, cervical, vaginal/vulvar, and penile cancers; and anogenital warts. For those patients who had attended the HIV clinic before January 1st 2006, medical records were reviewed for prior 2 years. All individuals with prevalent conditions at baseline and/or during the 2 years prior (for those with medical records as described above), were excluded from the study. Any clinical condition was recognized as an incident case only if the patients were free of the condition at baseline but developed it during follow-up. All clinical condition cases were verified by reviewing medical charts.

### Statistical analysis

Univariate analyses were conducted to compare demographic, sociobehavioral and clinical characteristics between patients with and without HPV-related CC during the follow-up period. All demographic (such as gender and race) and sociobehavioral (such as sexual orientation) information were self-reported. It is noted that 13.7% of participants did not self-report their sexual orientations and the data collection of sexual behavioral variables did not initiate since the beginning of our study period back in 2006. By contrast, all participants reported genders at any point of the study period. In order to retain the maximum number of cases, we decided to use self-reported gender variables instead of sexual behavioral variables. Chi-squared- and t-tests were used to compare categorical and continuous variables between the diseased and disease-free group, respectively.

Incidence rates (cases per 10,000 person-years) were computed for each HPV-related CC separately and compared between different sexes and races. Annual incidence for each condition was estimated followed by trend analyses using the Joinpoint Trend Analysis Software program (JTAS) [17]. Briefly, the Joinpoint regression model started with the minimum number of joinpoints and kept adding more until the number of joins was sufficient to distinguish between two unique and consecutive linear trends [17]. Monte Carlo permutation and Bayesian Information Criterion (BIC) were used for the goodness-of-fit test to find the best fitted curves over time [18]. The permutation method identified a time point that revealed an apparent change in trend. The final selected model comprehended the minimum number of joinpoints and smallest value of BIC.

The annual percent rate change (APC), with an assumption of a constant percentage change of the rate of the previous year was computed by the joinpoint regression. Incidence was log-transformed to diminish the effects of potential outliers in the linear regression. All APCs were then summarized to estimate an average annual percent change (AAPC) over a fixed interval [19]. The AAPC over any fixed interval was calculated using a weighted average of the slope coefficients of the underlying joinpoint regression line with the weights equal to the temporal length of each segment over the interval. The weighted average of slope coefficients was transformed to an annual percent change in the final step [20]. T-statistics were calculated for both APC and AAPC to assess the changes of slopes in the linear association.

Age-standardized incidence rates were initially estimated. The present study population was projected to the standard population of the 2016 US population from the Surveillance, Epidemiology, and End Results program (SEER) [21]. However, JTAS prohibits the calculation of age-adjusted rates with a dependent variable equal to zero cases under log transformation. Instead, crude incidence with a Poisson variance was used. Our main objective was to test whether there were any substantial changes on trends regarding incident HPV-related CC during the study period.

The study population consisted of less than 0.4% of self-reported transgender individuals (men to women), thus, the main analyses including incidence rate and trend comparisons were only conducted between men and women. Similarly, less than 4% of non-black or white participants (other races) presented in the study population, and hence, IR and trend analyses did not include the “other races”.

## Results

**Fig. 1:**
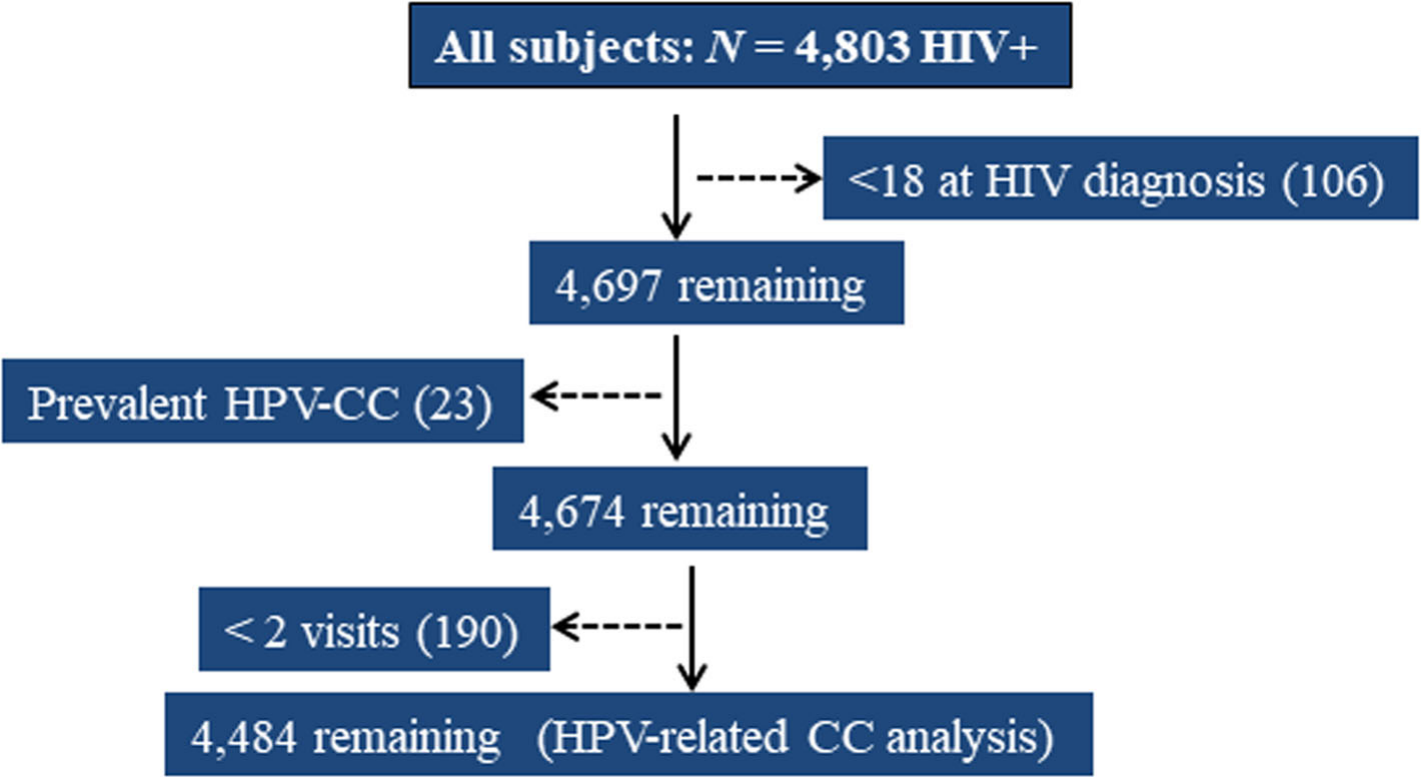
Study population flow chart of inclusion and exclusion criteria for all patients attending the HIV clinic between January 2006–March 2018

A total of 4803 PLWH attended the 1917 Clinic between January 1st 2006 and March 30th, 2017, however, 4484 patients met the inclusion/exclusion criteria (Fig. 1) with 3429, (76.5%) men, 1031 (23.0%) women, and 24 (0.5%) transgender individuals, 2676 (59.7%) blacks, 1632 (36.4%) whites, and 176 (3.9%) others. Among all the eligible participants, 1038 (23.1%) presented HPV-related CC (Table 1). The mean ages at the time of HPV-related CC presentation over the study follow-up were 41.8 (±10.6) years. The mean log10 VL (copies/mL) was higher in patients with HPV-CC compared to the non-case group, however the difference was not significant. The median nadir CD4 counts (cells/uL) were statistically higher in the non-cases (333, IQR: 139–853) than in patients with HPV-CC (237 [IQR: 72–701). Compared with women, men had much higher rates of HPV-related warts, anal LSIL, anal HSIL, and anal HPV-related cancer (*P* < 0.0001 for each comparison, Table 2). Overall, whites were more likely to be diagnosed with anal LSIL and cancer (*P* < 0.05, Table 2). Among women, whites were more likely to present cervical HSIL and cervical, vaginal and vulvar LSIL (*P* < 0.0001 for each comparison). HIV+ men had a higher rate than women to present anogenital warts (IR 190.4 vs 68.5 per 10,000 person-years), anal LSIL (188.2 vs 14.7 per 10,000 person-years), anal HSIL (43.2 vs 5.5 per 10,000 person-years), and anal cancer (25.8 vs 0 per 10,000 person-years) (Table 2). White men presented more frequently with anal LSIL and anal and penile cancers than black men (*P* < 0.03 for each comparison) (Table 2). MSM accounted for 54.9% of the total study population, and 62.5% of total HPV diagnoses made during the study period were attributed to them, with 14, 15.8, 2.8, 1.5, 0.17, and 0.12% diagnosed with warts, anal LSIL, anal HSIL, anal cancer, penile cancer, and Bowen’s disease, respectively.

**Table 1:** Demographic and clinical characteristics of the study population

Characteristics	Ever with HPV CC (*N* = 1038)	No-HPV CC (*N* = 3446)	*P*-value
Age at baseline^a^	38.3 (10.0)	41 (11.4)	< 0.0001
Age at HIV diagnosis ^a^	31.6 (8.8)	35.3 (10.7)	< 0.0001
Age at first HPV-CC^a^	41.8 (10.6)	–	–
Race			
Black	596 (57.4)	2079 (60.3)	< 0.0001
White	430 (41.4)	1203 (34.9)	
Others	12 (1.2)	164 (4.8)	
Median follow-up years	2.6 (0.9–5.3)	4.4 (1.9–8.0)	< 0.0001
Gender			
Male	779 (75.0)	2650	0.64
Female	249 (24.0)	782	
Transgender	10 (1.0)	14	
HIV risk factors			
MSM	646 (63.2)	1656	< 0.0001
Heterosexual	299 (29.2)	1384	
IVDU	77 (7.5)	300	
Others	1 (0.1)	5	
Mean Log VL (copies/mL)	5.3 (5.9)	5.2 (5.8)	0.59
Nadir CD4 (cells/μL)^b^	237 (72–701)	369 (0–899)	< 0.0001

^a^: mean (SD) are reported

^b^: median (25–75 IQR) are reported

**Table 2:** Incidence rates (IR) of HPV-related anogenital warts, Anal LSIL and HSIL in men and Cervical LSIL and HSIL in women

	# Cases	# Total	Person-years	IR (95%CI)	*P-*Value^‡^
HPV-related Clinical Conditions (by gender)^a^					
Anogenital warts	478	4484	29,646.2	161.2 (146.8–175.7)	–
Men	420	3429	22,286.2	188.5 (170.4–206.5)	< 0.0001
Women	51	1038	7507.9	67.9 (49.3–86.6)	
Anal LSIL	425	4484	21,854.0	194.5 (176.0–213.0)	–
Men	411	3429	21,842.3	188.2 (170.0–206.4)	< 0.0001
Women	8	1031	5436.6	14.7 (12.1–24.9)	
Anal HSIL	75	4484	22,195.0	33.8 (26.1–41.4)	–
Men	72	3429	16,665.5	43.2 (33.2–53.2)	< 0.0005
Women	3	1031	5434.8	5.5 (0–11.8)	
Anal cancer	43	4484	22,188.2	19.4 (13.6–25.2)	–
Men	43	3429	16,680.7	25.8 (18.1–33.5)	< 0.0001
Women	0	1031	5416.0	0	
Bowen’s disease	6	4484	22,232.1	2.7 (0.55–4.9)	–
Men	5	3429	16,701.2	3.0 (0.37–5.7)	0.66
Women	1	1031	5436.3	1.8 (0–5.6)	
HPV-related Clinical Conditions (by race)^a^					
Anogenital warts	478	4484	29,646.2	161.2 (146.8–175.7)	–
Black	312	2676	18,360.7	169.9 (151.1–188.8)	0.32
White	160	1632	10,382.2	154.1 (130.2–178.0)	
Anal LSIL	425	4484	21,854.0	194.5 (176.0–213.0)	–
Black	182	2676	12,892.1	141.2 (120.7–161.7)	< 0.0001
White	238	1632	8447.4	281.7 (207.8–317.5)	
Anal HSIL	75	4484	22,195.0	33.8 (26.1–41.4)	–
Black	40	2676	13,053.3	30.6 (21.1–40.1)	0.28
White	34	1632	8628.5	39.4 (26.5–53.3)	
Anal cancer	43	4484	22,188.2	19.4 (13.6–25.2)	–
Black	18	2716	13,030.0	13.8 (7.4–20.2)	0.025
White	24	1632	8643.7	27.8 (16.7–38.9)	
Cervical LSIL	171	1031	7352.3	232.6 (197.7–267.4)	–
Black	132	767	5679.6	232.4 (192.8–272.0)	0.69
White	38	236	1610.2	250.3 (102.1–290.9)	
Cervical HSIL	80	1031	7404.7	108.0 (84.5–131.7)	–
Black	54	767	5721.3	94.4 (69.2–119.6)	0.023
White	25	236	1529.5	163.5 (99.4–169.6)	
Cervical cancer	12	1031	5304.8	22.62 (9.8–35.4)	–
Black	6	767	4018.1	14.9 (3.0–26.9)	0.037
White	6	235	1203.4	49.9 (3.0–38.9)	
Vaginal/Vulvar cancer	15	1031	5304.3	28.3 (14.0–42.6)	–
Black	11	767	4018.1	27.4 (11.2–43.6)	0.74
White	4	236	1202.9	33.3 (0.67–65.8)	
Penile cancer	4	3429	16,493.2	2.3 (0.049–4.8)	
Black	3	886	8769.5	3.4 (0–7.3)	< 0.0001
White	0	1396	7298.4	0	
Bowen’s disease	6	4484	22,232.1	2.7 (0.55–4.9)	–
Black	3	2676	13,070.1	2.3 (0–4,9)	0.61
White	3	1632	8647.5	3.5 (0–7.4)	

^a^: Comparisons were only conducted between men vs. women and blacks vs. whites because very few transgender and other race individuals presented in the study

**Fig. 2:**
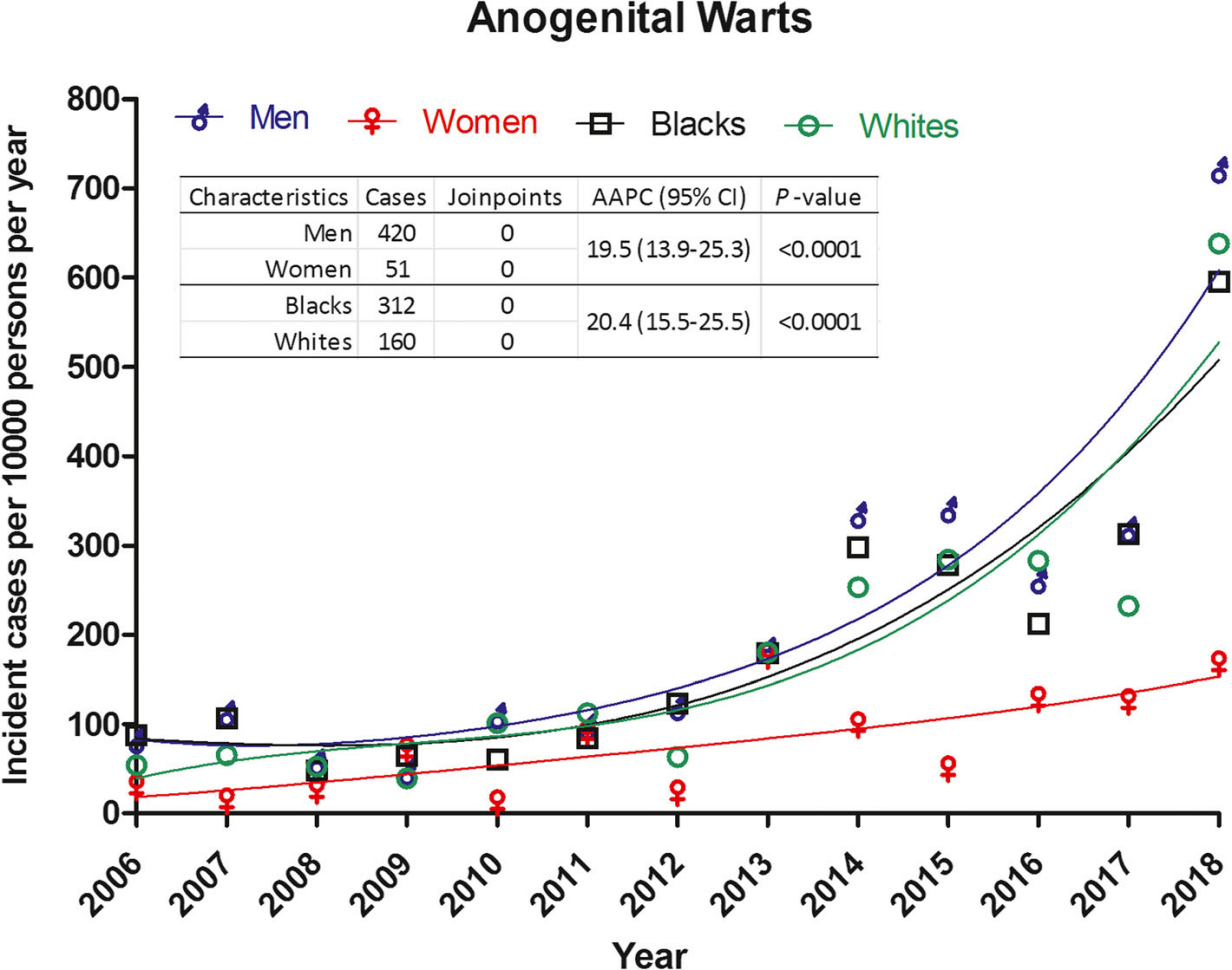
Trend of incident HPV-related anogenital warts stratified by genders and races between January 2006 and March 2018

**Fig. 3:**
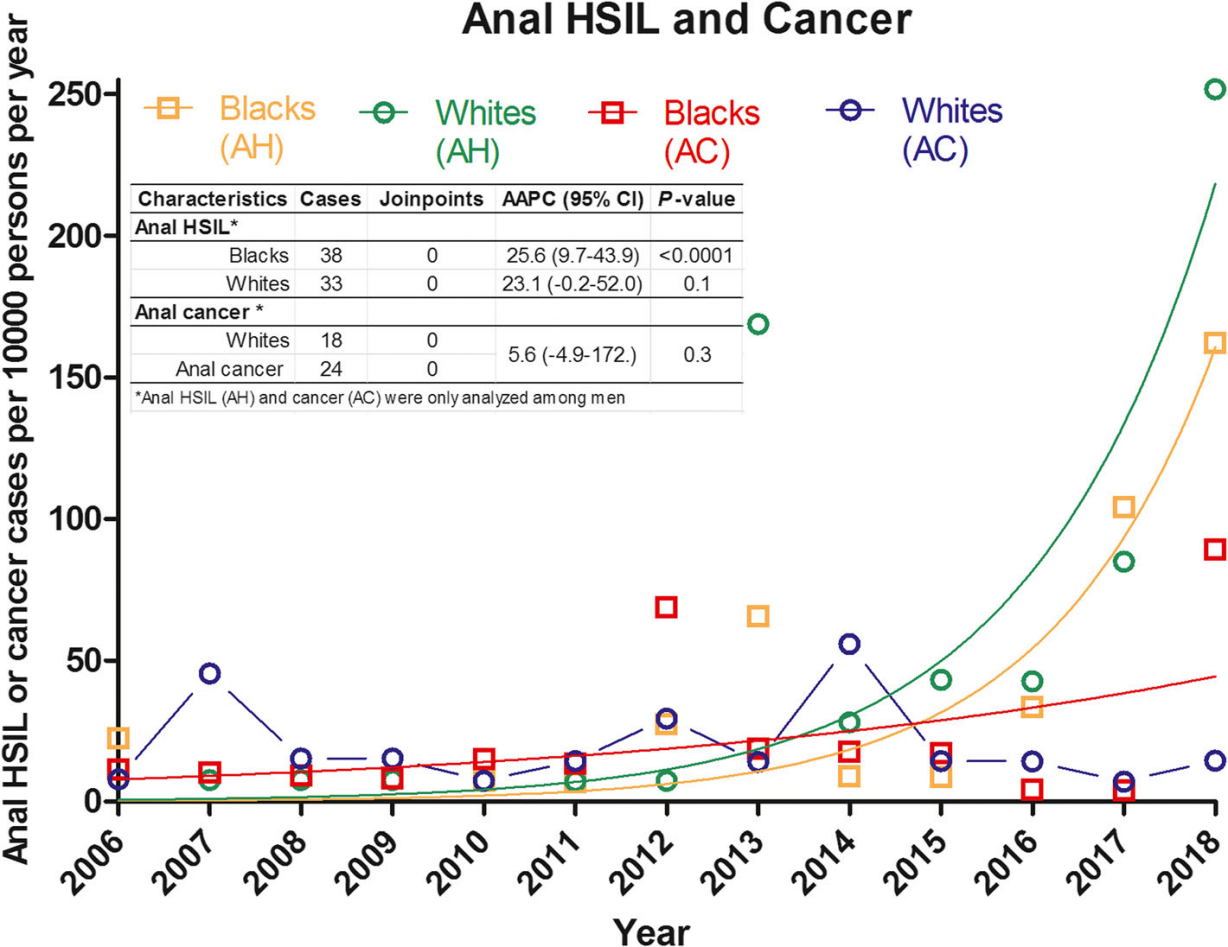
Trend of HPV-related incident anal HSIL and cancer stratified by genders and races between January 2006 and March 2018

HPV-related anogenital warts showed significant upward trends in both genders (AAPC: 19.5, *P* < 0.0001) and races (AAPC: 20.4, *P* < 0.0001) (Fig. 2). However, there were no distinct patterns between these two trends (*P* > 0.05 for test for parallelism, Table 3). The AAPC of anal HSIL among black men also showed an increasing incidence trend (AAPC: 25.6, *P* < 0.0001). Cervical HSIL and cancer did not show significant changes over time, but APC gave the joins that reflected periodic changes between 2006 and 2013 and 2016–2018 (*P* < 0.05 for each period) (Table 3, Fig. 3).

**Table 3:** Race and gender stratified trends of incident HPV-related anogenital warts, LSIL, HSIL, and cancer (men only), Cervical LSIL and HSIL, and cancer between 2006 and 2018

	Cases	Joins	Joinpoint Year	AAPC (95% CI)	*P*-Value
HPV-related CC					
Anogenital warts	–		–		
Men	420	0	–	19.5 (13.9–25.3)	< 0.0001
Women	51	0	–	19.5 (13.9–25.3)	< 0.0001
Black	312	0	–	20.4 (15.5–25.5)	< 0.0001
White	160	0	–	20.4 (15.5–25.5)	< 0.0001
Anal HSIL (men only)	71	0	–		
Black	38	0	–	25.6 (9.7–43.9)	0.0040
White	33	0	–	23.1 (−0.2–52.0)	0.052
Anal cancer (men only)	43	0	–	5.6 (−4.9, 17.2)	0.29
Black	18	0	–	–	–
White	24	0	–	–	–
Cervical HSIL		2	2013,2016	22.5 (−4.5–57.1)	0.11
Black	54	2	2013,2016		
White	25	2	2013,2016		
		–	2006–2013^*^	29.8 (11.6–50.9)	0.002
		–	2013–2016^*^	−38.3 (− 73.9–45.7)	0.25
		–	2016–2018^*^	179.6 (20.9–546.7)	0.020
Cervical cancer	12	2	2012, 2016	15.9 (−9.7–48.7)	0.2
Black	6	2	2012, 2016	–	–
White	6	2	2012, 2016	–	–
		–	2006–2012^*^	17.1 (−2.4–40.6)	0.084
		–	2012–2016^*^	−26.4 (− 58.7–31.3	0.27
		–	2016–2018^*^	177.5 (1.3–660.0)	< 0.0001

## Discussion

The present study indicate that HPV-related CC, particularly anal lesions and cancer were observed more in HIV+ men than women in the region. The incidence rate of anogenital warts constantly increased over the study period. Although cervical HSIL did not have monotonic trends, significant periodic increases in trends were detected. Although crude rates were reported, we computed the age-adjusted rates for warts, which did not generate 0 case over the follow-up. The alternative rates were similar to our results (data not shown). These observations and trends have never been reported in a clinical setting and this study with sufficient follow-up provides the broader scenario of these conditions among PLWH in the area. Our findings attempt to help clinicians better understand the burden of these comorbidities and drive better care in clinical settings.

HPV-related conditions were observed predominantly in men as compared to women. HIV+ men had almost a 3-fold greater risk of anogenital warts compared with women (Table 2), with no racial disparity observed. The trend of warts, however, increased approximately 20% each year (Table 3) regardless of gender and race. HIV+ men were also 8 and 25 times more likely to be diagnosed with anal HSIL and cancers, respectively, than HIV+ women (Table 2). However, in the general US population, HPV-related anal lesions and cancers are observed more in women than men [22]. There is a huge gap in screening guidelines for non-AIDS defining comorbidities, such as HPV-related anal precancerous lesions and cancers. Only women are currently screened for anogenital HPV-infection through the cervical cancer screening program [23]. The present study consisted of 76% men with limited anal cancer screenings. MSM were particularly susceptible for HPV-related CC. MSM are known to have an elevated risk of HIV acquisition. HIV+ MSM tend to be more likely infected with other STIs, such as HPV [24]. The study population consisted of 54% MSM, while they contributed to 62.5% total incident HPV diagnoses during the study period. That accounted for 72.2, 81.4, 92, 83.7, 100, and 50% of warts, anal LSIL, HSIL, and cancer, penile caner, and Bowen’s disease, respectively.

One of the largest HIV cohort, Multicenter AIDS Cohort Study (MACS), reported an overall incidence rate of anal cancer of 7 per 10,000 person-years among HIV+ MSM between 1984 and 2006 [25]. The finding from our study was over 3-fold greater than that rate (IR = 25.8 per 10,000 person-years among men) between 2006 and early 2018. In spite of the better immune status of PLWH in our cohort compared to MACS, specifically before ART regimen in 1996 [25], the median nadir CD4 counts were still significantly lower in patients with HPV-related CC than the non-cases (Table 1). Further, the rates of anal lesions and cancers increased exceptionally compared to the MACS. Geographically, the MACS, which predominantly includes white MSM, did not include a site in the Deep South of the US, and our findings provide evidence of higher trend in our self-reported MSM sub-population. Overall, there have not been many studies conducted among black MSM in the south regarding HIV and HPV comorbidities.

Although, we did not observe a monotonic trend of cervical HSIL or cancer, we were able to identify the periodic changes. For example, both conditions seemed to be growing in numbers of new diagnoses between 2016 and 2018 (Table 3) in both races. However, we have to take the screening programs implemented into account. In March 2016, the US Health Resources and Services Administration issued new screening guidelines for cervical cancer among HIV+ women, which included both cytology pap smears and serologic testing [23]. As an academic clinic, the UAB 1917 Clinic actively advocates HPV-related screenings for HIV+ women. The implementation of the new screening program could temporarily boost the number of new diagnoses of HPV-related cervical lesions and cancers. However, it does not necessarily mean an increase in cervical HPV infections.

This southeastern US region bears a heavy public health burden of HIV and STDs [26]. According to the US CDC’s national statistics, the incidence rates of HPV-related CC in our study were much higher among PLWH than the general population [2]. Although HPV infection is not curable if it persists, interventions may alleviate symptoms and prevent the HPV-related neoplasia. We had a long clinical follow-up in this clinical cohort, which allowed us to estimate incidence rates, while most other studies were only able to report incident HPV-related CC as percentages of new cases among PLWH. We specifically used the Joinpoint regression analysis to examine trends, which enabled us to report the statistical significance of changes in trends as well as compare trends between different sexes and races. However, we are aware that crude incidence rates were used for trend analysis, because log transformed Joinpoint regression model was conducted. Although PLWH are at higher risks of HPV-related anogenital conditions compared to the general population [1], most of these HPV-related HSIL and cancers were still not common among them. In order to normalize the distribution of the diagnosed cases over the study period, a log transformed rate model needs to be adopted. However, we would only be allowed to the log transformed models if there were cases presented every year throughout the follow-up. The JTAS would automatically add 0.5 if certain years have 0 cases. However, this procedure may be hard to achieve if age-adjusted rate is expected, because the addition of 0.5 is not recommended by the program in such scenario [17].

It is important to note that clinical diagnoses were based on patient willingness to seek medical attention. Unlike cervical cancer screening, anogenital screenings and examinations, especially for warts are not routinely performed in most clinical settings and are primarily recommended by providers and thus could reflect potential bias. In addition, since most of these infection related conditions except for cervical cancer are non-AIDS-defining, and hence, the Ryan White funding program provides limited coverage in the clinic. While this can under-estimate the number of cases, with a pro-active screening approach in this academic clinic setting, our estimated incidence shows a substantially higher rate than the estimates from the previous HIV studies. Diagnoses of HPV-related anogenital conditions are often initiated from physical presentations of warts and lesions. It is common practice to make prompt diagnoses and immediate treatment for most HPV-related CC without testing for viral infections.

## Conclusions

Our findings show that PLWH are at higher risks for anogenital HPV-related CC. However, screening for these conditions is not routinely conducted in clinics. Additional studies in southeast and other regions of US would be helpful to understand the epidemiology and trend of these conditions in PLWH. The present study attempts to provide information about these understudied conditions to guide screening and prevention strategies in clinical practices.

## Data Availability

The datasets generated and/or analyzed during the current study are available upon request in https://www.uab.edu/medicine/1917cliniccohort/.

## Abbreviations

CC: Clinical condition
HIV: Human immunodeficiency virus
HPV: Human papillomavirus
PLWH: People living with HIV infection
